# Supplementary material for: The discovery of novel noncoding RNAs in 50 bacterial genomes
Source: Nucleic Acids Res. 2024 Apr 22;52(9):5152–65. doi: 10.1093/nar/gkae248 (PMC11109978; doi:10.1093/nar/gkae248)
Supplement: gkae248_Supplemental_Files [file gkae248_supplemental_files.zip › Narunsky 50 Genomes RNA Motifs Files.zip!/Publication/44-D_geothermalis/Figures/LRC-44-1-NC_008025_1_IGR_37_2419319_2419560.sto.pdf]

LRC-44-1-NC_008025_1_IGR_37_2419319_2419560.sto.r2r

5′-UCCCCCUCCUUUCUCAACCUCGUGYUGACCAYCAUGCGUGAAGACCGCGCACCAUGYCGUUUGCCAGGCCUGAACUGAACAUAUCACGCGCGCGCGGCCCGUUCUCCAGCGAGGAAACGAGCGGCGUUACAGGCCGAGCGUCACCAGAACCUGCCGAGGAUCAGGGUGGCCCGGCGCUGCGCUCGACUUGCCUGGGCG
